# The Influence of Low-Emission Mineral Additives as a Substitute for CEM II and CEM III Cement on the Properties of Cement Mortars

**DOI:** 10.3390/ma18245673

**Published:** 2025-12-17

**Authors:** Paweł Muzolf, Grzegorz Rogojsz, Tomasz Rudnicki

**Affiliations:** Faculty of Civil Engineering and Geodesy, Military University of Technology, 2 Gen. Sylwestra Kaliskiego Str., 00-908 Warsaw, Poland; pawel.muzolf@wat.edu.pl (P.M.); grzegorz.rogojsz@wat.edu.pl (G.R.)

**Keywords:** cement substitutes, mineral additives in mortar, microsilica, glass flour, basalt flour, glass granulate, carbon footprint

## Abstract

The main goal of the research was to determine whether it was possible to reduce the cement content in mortar without compromising strength parameters. This is crucial for reducing the carbon footprint associated with cement production. In this article, the authors presented the results of research evaluating the effect of selected mineral additives on the strength properties of standard mortar after 7, 28, and 56 days of curing. The analysis of the effect of mineral additives was performed for CEM II and CEM III cements and seven selected mineral additives: white microsilica, Mikrosill+ microsilica, limestone powder, glass powder, glass granulate, and basalt powder. The study considered the use of mineral additives at 10% and 20% by weight of cement as a substitute. During the analysis of the test results, it was observed that the use of white microsilica and Mikrosill+ at 10% and 20% increased strength by approximately 50% compared to the reference samples. Importantly, strength was 50% higher with a 20% reduction in cement content. A positive effect of additives on strength parameters was observed only for CEMII cement. In the case of CEMIII cement, mineral additives reduce compressive strength.

## 1. Introduction

The global economy has been developing rapidly in recent years, which generates a market need for research development at both the laboratory and implementation stages in the search for materials that are intended to partially replace cement.

The authors believe that the use of such materials may lead to the implementation of corrections and optimization of mortar and concrete mix components. Today, we already commonly use new-generation chemical additives and admixtures [1,2,3,4], which significantly improve both the properties of concrete mixes and also increase the strength and durability of hardened concrete. The area on which all the major scientific units and the market of construction companies are working is the optimization of the mixture composition in order to minimize the impact on the environment by using, among others, alternative materials as clinker substitutes or supplementary cement materials mainly in the form of fly ash, slag or silica fume, which was widely presented in [5]. The main direction of action is to significantly reduce the share of cement through the use of mineral additives that have binding properties, which reduces the negative impact on the environment by reducing the carbon footprint of concrete. Taking such actions allows for a significant reduction of CO_2_ emissions into the atmosphere, as cement consumption is estimated at 4 gigatons per year [6]. Portland cement production technology is characterized by the highest energy consumption [7,8], which causes significant CO_2_ emissions of about 0.9 tons per 1 ton of produced cement [9] and according to [10] this constitutes about 5% of the total anthropogenic CO_2_ emissions in the world. The main component responsible for the emission of concrete is cement, the production process of which is highly energy-intensive due to the process emissions of clinker production. Clinker process emissions result from the thermal decomposition of calcium carbonate CaCO_3_^temp^ > CaO + CO_2_. In clinker production, process emissions constitute 63% of the carbon footprint. Therefore, the most effective way to reduce the carbon footprint is to reduce the clinker content in cement through the use of mineral additives. Therefore, an important way to reduce CO_2_ emissions in the process of implementing construction contracts is to use blended cements, such as CEM II or CEM III, which is consistent with the technological process associated with the need to increase the share of mineral components such as fly ash and silica fume [11,12]. Mineral materials containing a high content of calcium oxide in the limestone calcination process contribute to reducing carbon dioxide emissions [13,14,15]. Unfortunately, the use of clinker substitutes reduces the early strength and limits the possibility of using limestone [16,17]. The most commonly used material, however, is limestone. Its ability to increase strength stems from its physical properties and its reaction with aluminum oxide, creating the CO_2_-AFm phase [18,19,20]. It is therefore important to seek mineral materials that, when used as a cement substitute, will not compromise the strength parameters of mortars or concretes. One of the most frequently used materials is zeolite. Its use as a cement substitute in amounts up to 20% does not significantly affect the physical and mechanical properties of cement mortars [11,21,22]. However, the use of fly ash in most cases is associated with a strength decrease of 5% to 15% [23,24,25,26]. It should also be noted that resources of materials such as fly ash or slag may be limited. Therefore, it seems reasonable to look for mineral materials formed directly from rock crushing. One such material is quartz flour. Unfortunately, it has a negative impact on the strength of mortars, but when combined with silica fume, it contributes to increased compressive strength [27,28]. The use of mineral additives in cement concrete also has a beneficial effect on the distribution of air voids in hardened concrete, which directly contributes to increasing the frost resistance of concrete and its compressive strength, which is the result of changing the micro-characteristics of porosity [29,30,31,32]. Thanks to the use of mineral additives, we can also improve the workability of the mixture [33,34,35], which is directly related to reducing the carbon footprint resulting from the process of placing and compacting the concrete. The authors of publications [36,37] demonstrated that the use of fly ash, blast furnace slag, and silica fume not only increases workability but is also a crucial component in optimizing the composition of high-performance concretes, especially UHPC (ultra-high performance concrete). These additives influence the sealing of the cement matrix, which significantly improves the properties and durability of cement concrete. The authors also verified the effect of selected mineral additives on determining resistance to alkaline reactions, as presented in publication [38]. It should also be noted that research is also being conducted on the effect of organic additives on the properties of mortars and cement slurries [39]. Such additives reduce compressive strength but have a positive effect on the thermal resistance and elasticity of mortars. After a thorough analysis of the literature, in which the effect of mineral additives very often depended on the type of binder used and the aggregate grain size, the authors decided to continue the previous research in which mineral additives were used in the form of condensed microsilica, Mikrosill+ microsilica, limestone flour, glass flour, basalt flour, glass granulate and fly ash in combination with CEM I [40]. These studies revealed a beneficial effect of microsilica and fly ash on compressive strength after 7, 28, and 56 days of curing. For the remaining additives, a decrease in compressive strength of 6% to 25% was observed. Therefore, the main objective of this work, and its novelty, is to determine the properties of cement mortars with various mineral additives, which constitute the main components in combination with CEM II and CEM III cements. Considering the authors’ experience, it can be assumed that when using CEM II and CEM III cements, their content in the mortar can be reduced without compromising strength parameters.

The use of mineral additives as cement substitutes without compromising strength parameters is crucial because it can reduce the consumption of cement, the production of which is characterized by significant CO_2_ emissions. Reducing the cement content in cement concrete by up to 20% will have a significant environmental impact and allow for the reuse of waste materials from other industries.

## 2. Materials and Methods

### 2.1. Materials

In order to evaluate the effect of individual mineral additives on the strength properties of standard mortar using CEM II and CEM III cement, in the experimental tests, as in [39], standard sand from KWARCMIX (Smardzewice, Poland) was used, which meets the requirements specified in the standard [33]. The binder used was cement class CEM II B-V 32.5R HSR from Cemex (Warsaw, Poland), hereinafter referred to as CEM II, and CEM III A 42.5N LH/HSR/NA from Górażdże, Poland, hereinafter referred to as CEM III. The parameters of the cements used are presented in Table 1.

**Figure 1:**
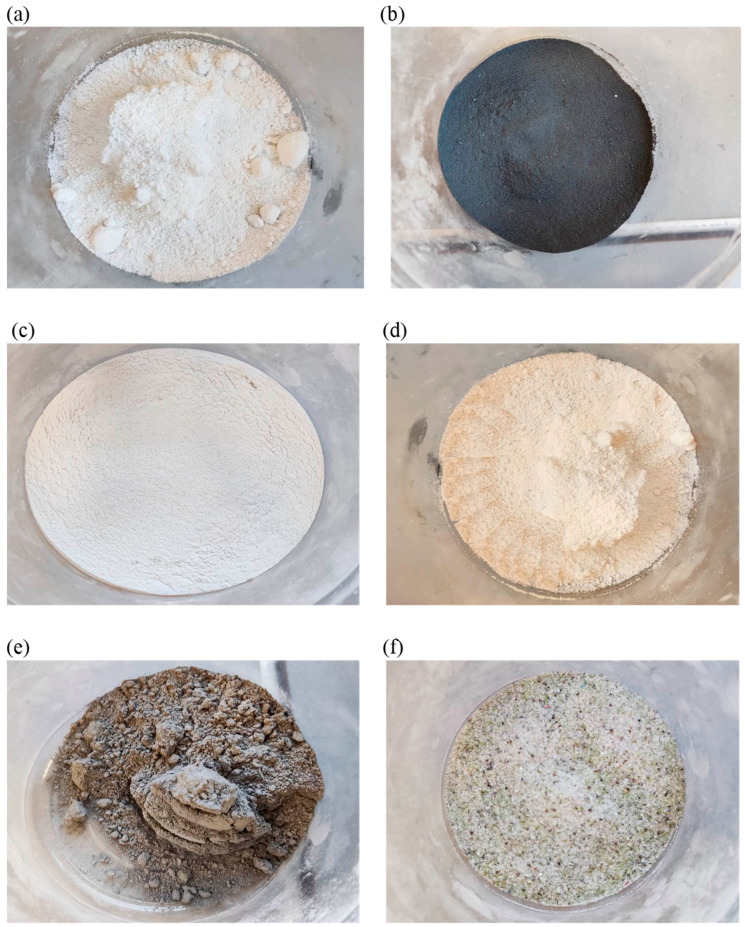
Mineral additives: (**a**) white microsilica; (**b**) Mikrosill+ microsilica; (**c**) glass flour; (**d**) limestone flour; (**e**) basalt flour; and (**f**) glass granulate.

The mineral additives used, as in [39], were white microsilica (MKb) and Mikrosill+ (MK+) from Mikrosilika Trade, in the form of silica dust generated in electric arc furnaces during the production of metallic silicon and ferrosilicon alloys. Another additive was limestone flour (MW), supplied by P.W. Sigma (Warsaw, Poland), commonly used as a filler for mineral-asphalt mixtures, resulting from the drying and grinding of limestone, whose main component is calcium carbonate. The effect of glass waste from P.P.H. REWA (Warsaw, Poland), produced on the basis of structural glass with a hardness of 6–7 on the Mosh scale in the form of glass flour (MS) and glass granulate (GS) with a grain size of 0/0.5 mm, was also examined. The next additive analyzed is basalt flour (PB) from NB Minerals, a waste product generated during aggregate grinding in mines. The chemical composition of individual additives is presented in Table 2, and the additives are shown in Figure 1. The chemical composition of mineral additives was developed based on the manufacturers’ declarations of properties.

### 2.2. Research Methods

Laboratory test samples were prepared according to the procedure described in EN 196-1 [41], in the form of prisms measuring 40 mm × 40 mm × 160 mm. After mixing the materials in a standard mixer, the samples were compacted in two layers in three-part molds on a shaking table with 60 impacts per layer. Nine samples were prepared for each additive, and three samples for each curing time, i.e., for 7, 28, and 56 days of mortar curing. As in [39], in the first stage of the study, 10% of the cement content was replaced with a mineral additive, and in the second stage, 20% of the cement content was replaced with a mineral additive. To compare the effects of individual additives and their contents, a reference mortar without mineral additives was prepared, consisting of 450 g of cement, 1350 g of standard sand, and 225 g of water.

Testing the basic characteristics of the designed mortars with mineral additives began by determining the consistency of each batch using a Vicat apparatus, equipped with a measuring rod with a diameter of 10.0 ± 0.05 mm and a measuring container with an internal diameter of 75 ± 10 mm and a height of 40.0 ± 0.2 mm. To determine the effect of mineral additives on mortar consistency, it was decided to test consistency in accordance with EN 196-3 [42]. Testing consistency in the Vicat apparatus allowed for a more precise determination of the effect of individual additives on mortar consistency than the classical method using a flow table.

Strength tests of the prepared mortars began by determining the flexural and compressive strength of the designed mortar samples. This test was performed on previously prepared prisms measuring 40 mm × 40 mm × 160 mm, and compressive strength tests were performed on the prism halves remaining after the flexural strength test. Bending and compressive strength tests were performed according to the procedure specified in EN 196-1 Cement testing methods—Part 1: Determination of strength. Both tests were conducted after 7, 28, and 56 days of curing of the mortar samples.

The composition of the mixes for stages 1 and 2 is presented in Table 3, and the description of the symbols used for the test samples is presented in Table 4. For 10% mineral additive content, the sample symbols contain the number 1 at the end, while samples with 20% mineral additive content contain the number 2 at the end.

## 3. Results

### 3.1. Marking the Consistency of Standard Mortar

**Figure 2:**
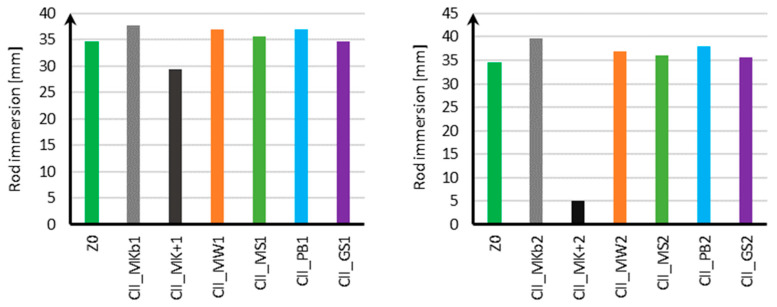
Consistency measurement results for CEM II.

**Figure 3:**
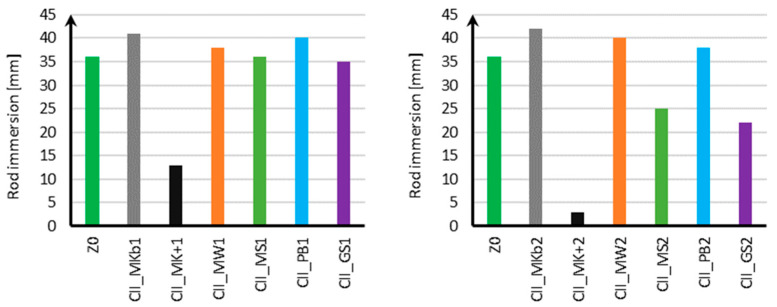
Consistency measurement results for CEM III.

The results of measuring the consistency of standard mortar with mineral additives in the amount of 10% and 20% for samples using CEM II cement are shown in Figure 2, and for samples using CEM III cement in Figure 3.

Based on the results obtained for samples using CEM II cement, it can be concluded that with the addition of 10% and 20% Mikrosill+ microsilica (CII_MK+1, CII_MK+2), the mortar consistency decreased from 35 mm for the standard mortar to 29 mm for 10% Mikrosill+ microsilica and up to 5 mm for 20% Mikrosill+ microsilica. For the remaining additives, the mortar consistency was similar to the standard mortar. The mineral additives used, apart from Mikrosill+ microsilica, did not negatively impact the mix’s compactability.

Analyzing the consistency results for samples using CEM III cement, it should be noted that, similarly to samples using CEM I cement, the addition of Mikrosill+ microsilica in amounts of both 10% and 20% caused a significant decrease in mortar consistency from 36 mm for the standard mortar to 13 mm for a 10% addition of Mikrosill+ microsilica and to 3 mm for a 20% addition of Mikrosill+ microsilica. In the case of samples using CEM III cement, the consistency of the mix did not change significantly with 10% of the remaining mineral additives. It should be noted that a 10% addition of white microsilica and basalt flour slightly increased the consistency of the mix from 36 mm to 41 mm for white microsilica and 40 mm for basalt flour, respectively. With a 20% content of mineral additives, a difference in the consistency of the mix is already visible. The mixture containing white microsilica showed the greatest increase, at 42 mm. The addition of limestone flour also showed a 4 mm increase in consistency compared to the mixture without the additives. The addition of glass flour and glass granulate resulted in a decrease in consistency of 9 mm for the glass flour and 14 mm for the glass granulate.

Based on the conducted research and the results of work [40], it can be concluded that the results of the mortar consistency are influenced not only by the mineral additives used and their content but also by the cement used. In the case of tests on the effect of mineral additives on standard mortar using CEM I cement, it was observed that with the addition of 10% and 20% microsilica, the mortar consistency decreased significantly from 32 mm for the mortar without additives to 10 mm for 10% compacted microsilica and 2 mm for 20% compacted microsilica. However, in the case of 10% limestone and glass flour content and 20% basalt flour content, the consistency was similar to that of the mortar without additives. In the conducted tests [39], it was observed that in general, with increasing mineral additive content, the consistency of the mixture decreases. The exception was the 20% basalt flour content, for which the consistency increased by approximately 10 mm. When using CEM I cement, the most beneficial effect on consistency improvement was observed with a 20% addition of basalt flour, which increased consistency by 10% compared to the mixture without additives. When using CEM II cement, the most beneficial effect was observed with a 20% addition of white microsilica, which increased consistency by 14%. When using CEM III cement, the most beneficial effect was observed with 10% and 20% additions of white microsilica. In both cases, the consistency increased by approximately 14%.

### 3.2. Flexural Strength

Flexural strength tests of mortar samples using CEM II and CEM III cement were performed after 7, 28, and 56 days of curing. The flexural strength test results for samples using CEM II cement are presented in Table 5, and for samples using CEM III cement in Table 6.

Analyzing the obtained results, it should be noted that it is not possible to clearly determine the effect of mineral additives and their content on flexural strength. At the same time, a decrease in flexural strength was observed for all additives. In the case of strength after 7 days of curing, the largest decrease in strength, 34%, was observed for 20% glass granulate content (CII_GS2), and the smallest, 2.6%, for 20% Mikrosill+ microsilica (CII_MK+2). In the flexural strength test after 28 days, the largest decrease in strength, 23.8%, was observed for samples with 20% limestone flour content (CII_MW2), and the smallest, 11.8%, for samples with 20% white microsilica (CII_MKb2). In the case of the test after 56 days of maturation, the highest decrease in flexural strength of 21.2% was observed for 20% glass granulate content (CII_GS2), and the lowest decrease of 3.2% for 20% limestone flour content (CII_MW2).

Analyzing the results obtained for samples using CEM III cement, it should be noted that the effect of mineral additives and their content on the flexural strength of the mortar samples cannot be clearly determined in this case either. In the case of strength after 7 days of curing, only samples with 20% Mikrosill+ microsilica (CIII_MK+2) and 20% limestone flour (CIII_MW2) achieved flexural strength greater than samples without additives by 4.8% and 8.4%, respectively. The remaining additives exhibited lower strength than samples without additives. Among them, samples with 20% glass granulate (CIII_GS2) performed the weakest, exhibiting 14.7% lower strength. However, in the flexural strength test after 28 days of curing, most mineral additives exhibited greater strength than samples without mineral additives. Among them, the highest flexural strength was achieved by samples with 20% white microsilica (CIII_MKb2) and 20% limestone flour (CIII_MW2), which were 21.9% higher than the strength of samples without additives. The lowest strength was achieved by samples with 10% and 20% glass granulate (CIII_GS1, CIII_GS2), whose strength was 13.1% and 10.8% lower, respectively. In the case of flexural strength testing after 56 days of curing, most of them achieved a strength lower than the samples without additives. The lowest strength was achieved by samples with 20% basalt flour (CIII_PB2), whose strength was 15.2% lower than the samples without additives. Samples with higher strength than those without additives include those with 10% white microsilica (CIII_MKb1), limestone flour (CIII_MW1), and glass granulate (CIII_GS1), as well as those with 20% white microsilica (CIII_MKb2) and limestone flour (CIII_MW2). These samples have the highest strength, which is 6% higher than those without additives.

### 3.3. Compressive Strength

**Figure 4:**
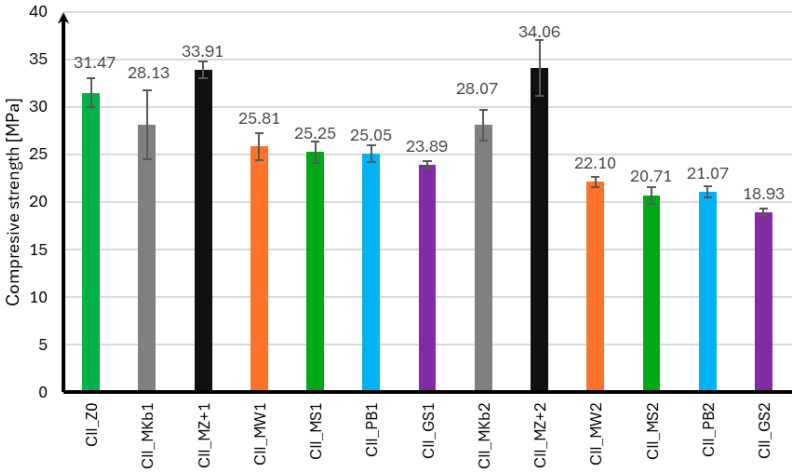
Compressive strength test results after 7 days for CEM II.

**Figure 5:**
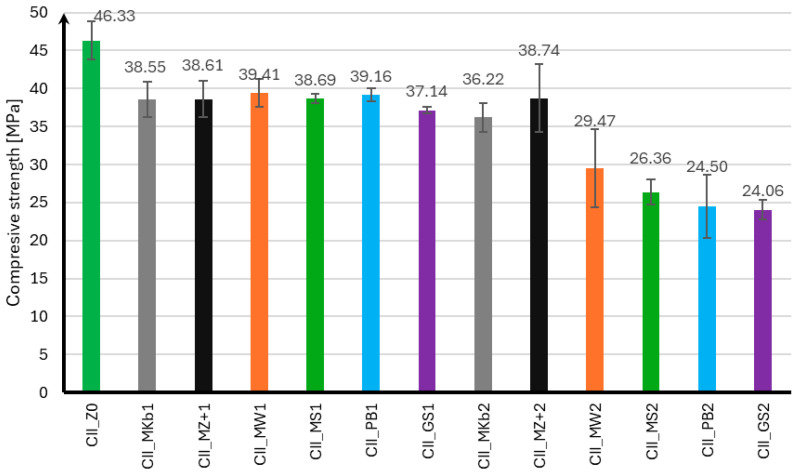
Compressive strength test results after 28 days for CEM II.

**Figure 6:**
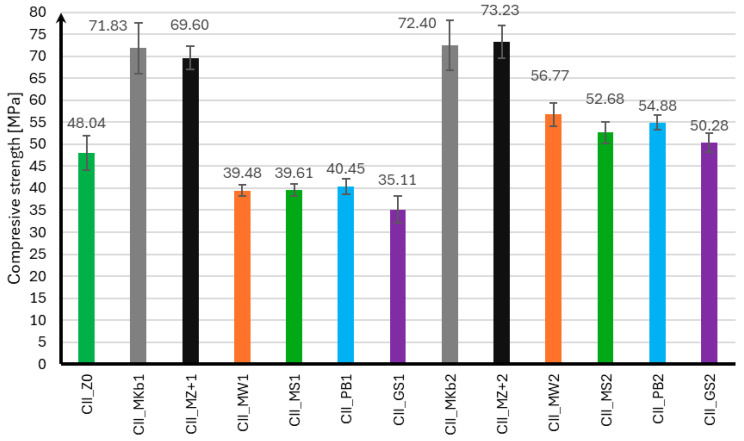
Compressive strength test results after 56 days for CEM II.

**Figure 7:**
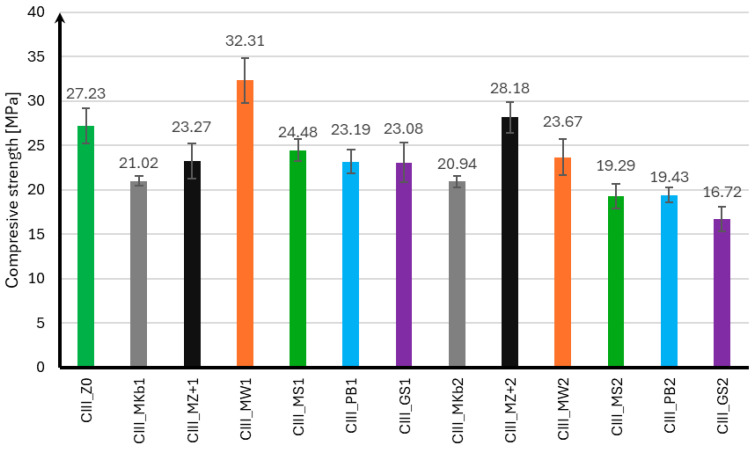
Compressive strength test results after 7 days for CEM III.

**Figure 8:**
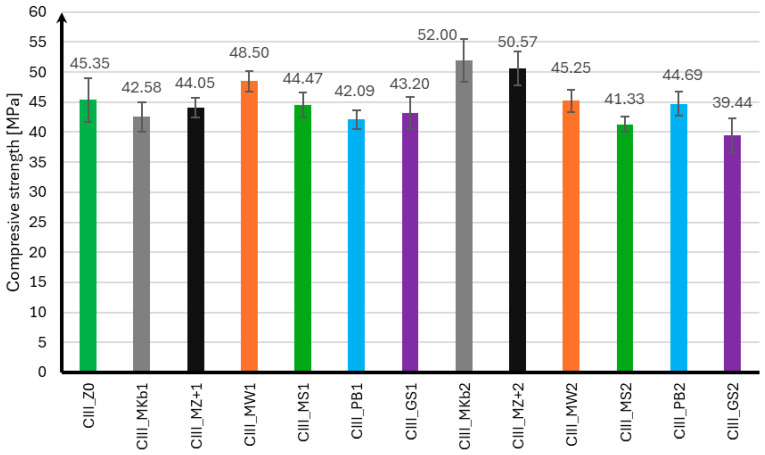
Compressive strength test results after 28 days for CEM III.

**Figure 9:**
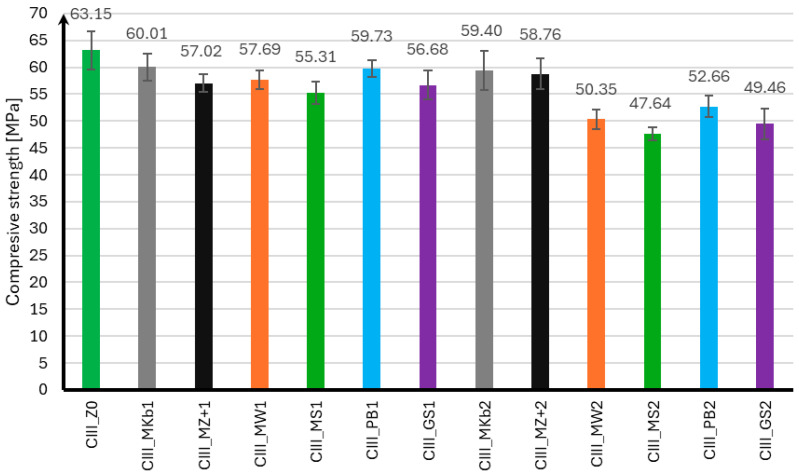
Compressive strength test results after 56 days for CEM III.

The compressive strength of mortar samples using CEM II and CEM III cements was performed in the same manner as for the flexural strength test after 7, 28, and 56 days of curing. The compressive strength results for samples using CEM II cement are presented in Figure 4, Figure 5 and Figure 6, and for samples using CEM III cement in Figure 7, Figure 8 and Figure 9.

Analyzing the presented compressive strength results for samples using CEM II cement, it should be noted that mineral additives adversely affect the obtained compressive strength in the initial curing phase. Only samples with 10% and 20% Mikrosill+ microsilica added achieved compressive strengths after seven days that were higher than those without additives. At the same time, it can be observed that samples with 20% additives had slightly lower strength than those with 10% additives. After 28 days of curing, most samples with mineral additives achieved similar strengths and were approximately 17% lower than those without additives. The exceptions are samples with 20% limestone flour, glass flour, basalt flour, and glass granulate, whose compressive strengths were 36%, 43%, 47%, and 48% lower than those without additives, respectively. It should also be noted that samples with 10% and 20% of Microsill+ microsilica, which had the highest strength after seven days, achieved the lowest strength increase after 28 days of curing. The situation is completely opposite when analyzing the results after 56 days of curing. Samples with the lowest strength after 28 days of curing achieved a significant increase in strength, exceeding 100% compared to the strength after 28 days of curing. It should also be noted that samples with a 10% addition of limestone flour, glass flour, basalt flour, and glass granulate showed virtually no increase in strength compared to the strength after 28 days of curing. Samples with 10% and 20% white microsilica and Mikrosill+ microsilica showed the highest strength. Their strengths are on average about 50% higher than the strength of samples without additives.

In the case of the compressive strength results for samples made using CEM III cement, it can be concluded that the use of mineral additives did not positively affect the strength of the samples. Analyzing the strength results after seven days of curing, only samples with 10% limestone flour achieved strength greater than samples without additives by 19%. The remaining mineral additives were characterized by strength lower than samples CIII_MS1 by 10% and CIII_GS2 by 39%. It can also be noted that samples with 20% Mikrosill+ microsilica achieved strength at the same level as the base samples. After 28 days of curing, only samples with 10% limestone flour and 20% white microsilica and Mikrosill+ had strength greater than samples without additives by 7%, 15%, and 12%, respectively. The remaining samples have lower strengths, but similar to those of the base samples, except for samples with 20% glass flour and glass granulate, which had 9% and 13% lower strengths. Unfortunately, the strength results after 56 days of curing show that all mineral additives have lower strengths than the base samples. The compressive strength of most mineral additives ranges from 55 MPa to 60 MPa, which is similar to the strength of the samples without additives, which is 63.15 MPa. The exceptions are samples with 20% limestone flour, glass flour, basalt flour, and glass granulate, whose strengths are 20%, 25%, 17%, and 22% lower than the samples without additives, respectively.

**Figure 10:**
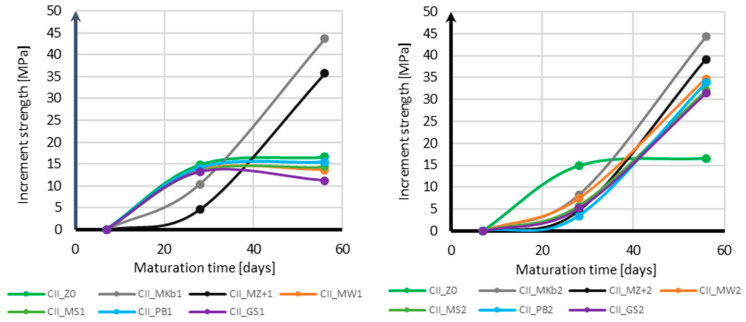
Increase in compressive strength over time for CEM II.

**Figure 11:**
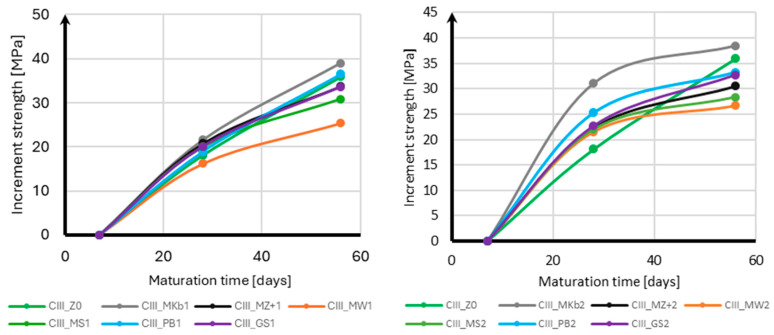
Increase in compressive strength over time for CEM III.

Based on the compressive strength tests, an analysis of the increase in compressive strength over time was made for each additive, depending on its content, divided into samples with CEM II and CEM III cement. Figure 10 presents the results of the increase in compressive strength over time for samples with CEM II cement, and Figure 11 for samples with CEM III cement.

Analyzing the strength gain over time for samples with CEM II cement, it should be noted that in the case of samples with 10% mineral additives, the strength gain stopped after 28 days of curing, even in samples without mineral additives. The only exceptions are the samples with white microsilica and Mikrosill+ additives, which are characterized by an increased strength gain between 28 and 56 days of conditioning. Their strength gain between 28 and 56 days of conditioning is approximately four times greater than between 7 and 28 days of curing. The situation is completely different for 20% additives. In this case, all samples with mineral additives are characterized by a greater strength gain between 28 and 56 days of conditioning, which for all additives is approximately four times greater than between 7 and 28 days of conditioning.

When using CEM III cement, analyzing the strength gain over time for samples with 10% mineral additives reveals a uniform strength gain over time for both the samples with mineral additives and the base samples. All samples exhibit similar strength gains between days 7 and 28 of conditioning and between days 28 and 56 of conditioning. For 20% mineral additives, a significant decrease in strength gain is observed between days 28 and 56 of conditioning for all mineral additives. The strength gain between days 28 and 56 of conditioning in this case is approximately four times smaller than between days 7 and 28 of conditioning.

### 3.4. Cost–Benefit Analysis

To determine the benefits of using mineral additives in cement mortars, a cost–benefit analysis was conducted. Table 7 summarizes the prices of each mineral additive and the CEM II and CEM III cements used. Table 8 presents the costs of preparing mortar samples, divided into samples with CEM II and CEM III cement, and for each mineral additive content. Because the aggregate content was the same in each sample, its cost was omitted from the comparison. The table also shows the increase in mortar price compared to the mortar without additives, for comparison to samples with CEM II and CEM III cement. The percentage increase in final compressive strength is also presented, i.e., after 56 days of sample conditioning.

Based on the analysis of the prices of the materials used, it can be concluded that all mineral additives, except limestone flour, are several times more expensive than cement. The most expensive of these is white microsilica, whose price is approximately 19 times higher than cement. The most favorable price, however, is limestone flour, whose price is approximately half that of cement.

Analyzing the presented results, it can be concluded that the largest price increase is observed for samples with the addition of white and Mikrosill+ microsilica. This increase is approximately 190% and 380% for the addition of white microsilica, and approximately 90% and 180% for the addition of Mikrosill+ microsilica. A similar price increase was observed for samples with both CEM II and CEM III cement. It should also be noted that in the case of samples with CEM II cement, the increase in the cost of manufacturing samples with the addition of both microsilicas is associated with a simultaneous increase in compressive strength of approximately 50%, at 10% and 20% of their content. The 20% addition of limestone flour is also noteworthy. In this case, a 10% reduction in the sample manufacturing cost resulted in an 18% increase in compressive strength. In the case of a 10% limestone flour content, a 5% price reduction resulted in an 18% decrease in strength compared to samples without the additives. It should also be noted that for samples with CEM II cement, the least favourable effect was achieved by using 10% and 20% glass flour addition. In the first case, with a 58% cost increase, an 18% decrease in compressive strength was observed. In the second case, with a 20% glass flour content and a 116% cost increase, only a 10% increase in strength was observed. The use of a 20% basalt flour additive also proved beneficial, with an 8% cost increase and a 14% increase in compressive strength. Unfortunately, in the case of CEM III cement, the use of additives negatively impacts both price and strength. In this case, no favorable increase in sample production cost was observed that would translate into increased strength. A significant decrease in strength was observed for all samples, which, given cost increases exceeding 360%, is unfavourable.

The aim of the study was to determine the potential for reducing the carbon footprint of mortar by using low-emission mineral additives as a substitute for CEM II and CEM III multi-component cements. These cements differed in clinker content and the type and amount of mineral additives in their composition, in accordance with the requirements of the EN 197-1 standard. For LCA (Life Cycle Assessment) calculations in accordance with the ISO 14067 standard [43], the actual transport distances of cement, aggregate, and mineral additives to the construction site were assumed, amounting to 80, 100 and 120 km, respectively. After calculating CO_2_ emissions for individual components of the concrete mix and knowing the distance from the construction site, the determined carbon footprint after replacing cement with 10% and 20% mineral additives is presented in Table 9.

Analysis of results obtained using software recommended by the Global Cement and Concrete Association (GCCA) shows that using CEM II cement with 20% mineral substitution reduces the carbon footprint by 32.40%, while using CEM III cement with 20% mineral substitution in mortars for building components resulted in a 53.12% reduction. This is a significant result, suggesting that the wider use of cements with mineral additives in construction should be considered.

## 4. Discussion

Analyzing the results of the tests conducted to determine the consistency of the mortars, it can be concluded that in both CEM II and CEM III cements, the addition of Mikrosill+ microsilica negatively affects the consistency of the mortar. Its 20% content contributes to a significant decrease in consistency, which significantly impedes compaction of the mixture. In the case of CEM II cement, the 20% addition of glass flour and glass granulate also contributed to a significant decrease in the consistency of the mortar. The remaining mineral additives, at both 10% and 20% concentrations for both cements, do not adversely affect the workability of the mortar and even improve its consistency, suggesting that it is possible to reduce the amount of mixing water and thus potentially increase compressive strength. Similar research results were obtained in a study analyzing the effect of mineral additives on the properties of mortar using CEM I cement [40].

The effect of mineral additives cannot be clearly determined based on flexural strength determinations. The studies yielded varying results depending on the mineral additive content and the cement used. It should be undoubtedly stated that in the case of using CEM II cement, all mineral additives at their 10 and 20% content caused a slight decrease in flexural strength compared to samples without additives, which was within the limits of measurement error. However, in the case of CEM III cement, some of the additives had a positive effect on strength parameters, while others had a negative effect. It should also be noted that a 10% content of mineral additives does not significantly affect the final strength, except for basalt flour, which significantly reduces flexural strength. A greater difference in the results is already visible for a 20% content of mineral additives. In this case, the beneficial effect of limestone flour and white microsilica is clearly visible, which increase flexural strength after 56 days of conditioning.

A different effect of additives was observed when analyzing the compressive strength results. In the case of CEM II cement, only samples with 10% limestone flour, glass flour, basalt flour, and glass granulate content showed lower strength than samples without additives. The remaining mineral additives had a positive effect on the strength parameters. In the case of limestone, glass and basalt flour, which are not considered mineral additives exhibiting pozzolanic reactivity with cement, due to their specific surface area they increased the tightness of the mixture, which translated into an increase in compressive strength. It should also be noted that the use of additives in the form of white microsilica and Mikrosill+ contributed to an increase in strength by approximately 50% compared to samples without additives. Importantly, a 50% higher strength was achieved with a 20% reduction in cement content. In the case of CEM III cement, no beneficial effect of mineral additives on the final compressive strength was observed. Each of the mineral additives used contributed to a decrease in strength to a greater or lesser extent.

Similar results can be obtained by analyzing the literature, in which the authors investigated the effects of various mineral additives on the parameters of standard mortars and cement concretes. In the results presented in [44], the authors observed a beneficial effect of fly ash only after 96 days of sample conditioning. Similarly, in the case of microsilicas, the results in [45] showed strength decreases ranging from 19% to 35% compared to standard samples. Similar results were presented in the study on the effect of glass flour [46], in which the authors observed a strength decrease of 16%.

Taking into account the authors’ research results regarding the effect of mineral additives on standard mortar using CEM I [40] cement, it should be noted that the suitability of a given mineral additive cannot be unequivocally determined. However, it is certain that it is possible to reduce the cement content in the mortar and replace it with waste material without risking loss of strength parameters.

## 5. Conclusions

Analyzing the obtained test results to determine the effect of selected mineral additives on the properties of standard mortar using CEM II and CEM III cements, the following conclusions can be drawn:

1. It t is possible to replace CEM II B-V 32.5R HSR cement with mineral additives in the form of white and Mikrosil+ microsilica in the amount of 10 and 20% without the risk of losing strength parameters.
2. It is possible to replace CEM II B-V 32.5R HSR cement with mineral additives in the form of limestone flour, glass flour, basalt flour and glass granulate in the amount of 20% without the risk of losing strength parameters.
3. The analyzed mineral additives, such as microsill+, glass flour, and glass granulate, negatively affect the consistency of the cement mortar. However, the remaining tested mineral additives increase consistency.
4. For both CEM II and CEM III cements, no effect of mineral additives on the flexural strength was observed compared to the standard mortar.
5. The use of 20% microsill+ and white microsilica resulted in a 50% increase in compressive strength despite a 20% reduction in CEM II cement.
6. In the case of CEM III cement, mineral additives significantly reduce compressive strength.

In addition to the numerous advantages of using mineral additives as a cement substitute, it’s also important to remember the limitations associated with their availability, high cost, and the need to adapt current production technologies. The use of additional materials in concrete production will require the installation of silos and feeders in existing facilities.

Further development work should also determine the possibility of using mineral additives in concrete used in industrial construction.

## Figures and Tables

**Table 1:** Properties of cement.

Property	Unit	CEM II	CEM III
Specific surface area	[cm^2^/g]	4787	4690
Start of setting time	[min]	220	199
End of setting time	[min]	283	274
Change in volume	[mm]	1.3	0.5
Compressive strength			
After 2 days	[MPa]	16.9	13.7
After 28 days	[MPa]	42.3	50.6
Contents SO_3_	[%]	2.84	2.06
Contents Cl	[%]	0.056	0.063
Share of silica fly ash	[%]	28.64	-
Loss of ignition	[%]	-	0.89

**Table 2:** Chemical composition of the additives used.

Component	Unit	MKb	MK+	MW	MS	PB	GS
SiO_2_	[%]	>80.0	>85.0	3.5	>65.0	38.2	>65.0
CaO	[%]	<3.5	<1.0		>8.0	15.2	>8.0
CaCO_3_	[%]	-	-	93.0	-	-	-
FeO_3_	[%]	-	-	0.3	<0.2	15.9	<0.2
MgO	[%]	-	-	0.7	<0.4	7.7	<0.4
SO_3_	[%]	<4.0	<2.0	-	-	0.2	-
Na_2_O	[%]	<8.0	<0.5	-	>14.0	2.9	>14.0
Al_2_O_3_	[%]	-	-	-	2.0	12.7	2.0
Cl^−^	[%]	<1.8	<0.3	-	-	0.07	-

**Table 3:** Recipes.

Research Stage	Aggregate Content [g]	Cement Content [g]	Contents of the Additive [g]	Water Content [g]
Control mix	1350	450	0	225
Stage 1 10% additives	1350	405	45	225
Stage 2 20% additives	1350	360	90	225

**Table 4:** Symbols of research samples.

Mineral Additives	Designation for CEM II	Designation for CEM III
White microsilica	CII_MKb	CIII_MKb
Mikrosill+ mikrosilica	CII_MK+	CIII_MK+
Limestone flour	CII_MW	CIII_MW
Glass flour	CII_MS	CIII_MS
Basalt flour	CII_PB	CIII_PB
Glass granulate	CII_GS	CIII_GS

**Table 5:** Flexural strength results for CEM II.

Mineral Supplement	Flexural Strength After 7 Days/Standard Deviation [MPa]	Flexural Strength After 28 Days/Standard Deviation [MPa]	Flexural Strength After 56 Days/Standard Deviation [MPa]
CII_Z0	5.70/0.11	8.48/0.39	10.19/0.49
CII_MKb1	5.23/0.15	7.38/0.38	9.57/0.19
CII_MKb2	4.80/0.17	7.48/0.12	9.53/0.29
CII_MK+1	5.27/0.10	7.05/0.57	9.74/0.55
CII_MK+2	5.55/0.22	7.14/0.63	9.81/0.16
CII_MW1	5.08/0.15	7.01/0.56	9.41/0.24
CII_MW2	4.38/0.11	6.46/0.60	9.86/0.13
CII_MS1	4.65/0.34	7.34/0.57	9.63/0.38
CII_MS2	4.10/0.29	6.59/0.38	9.82/0.34
CII_PB1	4.18/0.06	7.02/0.17	9.09/0.77
CII_PB2	4.30/0.11	6.79/0.39	8.69/0.10
CII_GS1	3.98/0.38	6.82/0.60	9.05/0.82
CII_GS2	3.75/0.51	6.80/0.28	8.03/0.43

**Table 6:** Flexural strength results for CEM III.

Mineral Supplement	Flexural Strength After 7 Days/Standard Deviation [MPa]	Flexural Strength After 28 Days/Standard Deviation [MPa]	Flexural Strength After 56 Days/Standard Deviation [MPa]
CIII_Z0	6.45/0.43	8.99/0.77	11.13/0.39
CIII_MKb1	5.95/0.38	9.66/0.48	11.45/0.39
CIII_MKb2	6.09/0.58	10.96/0.16	11.79/0.35
CIII_MK+1	5.78/0.09	9.59/0.45	11.01/0.49
CIII_MK+2	6.76/0.46	9.51/0.12	10.80/0.35
CIII_MW1	6.16/0.32	10.02/0.34	11.22/0.34
CIII_MW2	6.99/0.52	10.95/0.31	11.80/0.86
CIII_MS1	6.29/0.12	9.55/0.04	10.65/0.32
CIII_MS2	6.00/0.69	8.66/0.41	10.13/0.10
CIII_PB1	6.17/0.08	9.48/0.33	9.93/0.42
CIII_PB2	6.05/0.23	8.70/0.41	9.44/0.36
CIII_GS1	6.13/0.34	7.81/0.14	11.25/0.42
CIII_GS2	5.50/0.06	8.02/0.21	9.72/0.35

**Table 7:** Price of materials used.

Material	Cost [Zł/t]	Cost [EUR/Ton]
Cement CEM II	790	185.9
Cement CEM III	834	196.2
White microsilica	15,960	3755.3
Mikrosill+ microsilica	7960	1872.9
Limestone flour	380	89.4
Glass flour	5360	1261.2
Basalt flour	1100	258.8
Glass granulate	1200	282.4

**Table 8:** Costs of making mortar samples.

Samples	Price of Mortar Without Sand [Zł]	Price Increase	Increase in Compressive Strength After 56 Days
CII_Z0	0.36	-	-
CII_MKb1	1.04	192%	50%
CII_MZ+1	0.68	91%	45%
CII_MW1	0.34	−5%	−18%
CII_MS1	0.56	58%	−18%
CII_PB1	0.37	4%	−16%
CII_GS1	0.37	5%	−27%
CII_MKb2	1.72	384%	51%
CII_MZ+2	1.00	182%	52%
CII_MW2	0.32	−10%	18%
CII_MS2	0.77	116%	10%
CII_PB2	0.38	8%	14%
CII_GS2	0.39	10%	5%
CIII_Z0	0.38	-	-
CIII_MKb1	1.06	181%	−5%
CIII_MZ+1	0.70	85%	−10%
CIII_MW1	0.35	−5%	−9%
CIII_MS1	0.58	54%	−12%
CIII_PB1	0.39	3%	−5%
CIII_GS1	0.39	4%	−10%
CIII_MKb2	1.74	363%	−6%
CIII_MZ+2	1.02	171%	−7%
CIII_MW2	0.33	−11%	−20%
CIII_MS2	0.78	109%	−25%
CIII_PB2	0.40	6%	−17%
CIII_GS2	0.41	9%	−22%

**Table 9:** Carbon footprint reduction results in cement mortar using multi-component cements [9,11].

Parameter	Unit	CEM I	CEM II	CEM III
Carbon footprint cement	[CO_2_/kg]	0.703	0.559	0.377
Cement transportation	[CO_2_/km/t]	0.199	0.199	0.199
Carbon footprint granite aggregate	[CO_2_/kg]	0.007	0.007	0.007
Aggregate transport	[CO_2_/km/t]	0.166	0.166	0.166
Fly ash mineral additive	[CO_2_/kg]	-	0.140	-
Granulated Blast Furnace Slag additive	[CO_2_/kg]	-	-	0.140
Mineral additive transport	[CO_2_/km/t]	0.175	0.175	0.175
Carbon footprint of mortar in cubic meters	[CO_2_/m^3^]	316.35	251.55	169.65
Carbon footprint of mortar in cubic meters with a 10% cement reduction	[CO_2_/m^3^]	291.02	232.70	158.99
Carbon footprint of mortar in cubic meters with a 20% cement reduction	[CO_2_/m^3^]	265.68	213.84	148.32
Carbon footprint reduction to CEM I thanks to 20% reduction in cement volume	[%]	−16.02	−32.40	−53.12

## Data Availability

The original contributions presented in this study are included in the article. Further inquiries can be directed to the corresponding author.
